# Activation Complexity: A Cognitive Impairment Tool for Characterizing Neuro-isolation

**DOI:** 10.1038/s41598-020-60354-2

**Published:** 2020-03-03

**Authors:** Nicholas J. Napoli, Matthew Demas, Chad L. Stephens, Kellie D. Kennedy, Angela R. Harrivel, Laura E. Barnes, Alan T. Pope

**Affiliations:** 1 Industrial and Systems Engineering, University of Florida, Gainesville, FL 32611 United States; 2 Systems and Information Engineering, University of Virginia, Charlottesville, VA 22904 United States; 3 NASA Langley Research Center, Hampton, VA 23681 United States; 4 National Institute of Aerospace, Hampton, VA 23681 United States; 5 University of Florida, Dept. of Electrical and Computer Engineering, Gainesville, FL 32611 United States

**Keywords:** Hypoxic-ischaemic encephalopathy, Electroencephalography - EEG

## Abstract

Electroencephalography (EEG) is a method for recording electrical activity, indicative of cortical brain activity from the scalp. EEG has been used to diagnose neurological diseases and to characterize impaired cognitive states. When the electrical activity of neurons are temporally synchronized, the likelihood to reach their threshold potential for the signal to propagate to the next neuron, increases. This phenomenon is typically analyzed as the spectral intensity increasing from the summation of these neurons firing. Non-linear analysis methods (e.g., entropy) have been explored to characterize neuronal firings, but only analyze temporal information and not the frequency spectrum. By examining temporal and spectral entropic relationships simultaneously, we can better characterize how neurons are isolated, (the signal’s inability to propagate to adjacent neurons), an indicator of impairment. A novel time-frequency entropic analysis method, referred to as Activation Complexity (AC), was designed to quantify these dynamics from key EEG frequency bands. The data was collected during a cognitive impairment study at NASA Langley Research Center, involving hypoxia induction in 49 human test subjects. AC demonstrated significant changes in EEG firing patterns characterize within explanatory (p < 0.05) and predictive models (10% increase in accuracy). The proposed work sets the methodological foundation for quantifying neuronal isolation and introduces new potential technique to understand human cognitive impairment for a range of neurological diseases and insults.

## Introduction

Electroencephalography (EEG) detects the electrical activity of the brain and analysis of EEG permits tracking variations in brain wave patterns. EEG analysis provides information about a person’s cognitive state such as response inhibition, level of concentration, arousal, and even diagnostic information regarding diseases such as Alzheimer’s, post-cardiac arrest syndrome (hypoxic encephalopathies), and epilepsy^1–6^. There are many types of analyses designed to extract features from EEG signals that examine coherence, intensity of frequency bands, signal entropy, coupling, and source localization to acquire information about cognitive states^2,7^. These extracted EEG features are then used as the foundation for explanatory and predictive modeling. Typically, two or more of these features are utilized to generate a feature space for predictive models that can predict epilepsy, hypoxia, etc.^2^. Thus, capturing these new EEG features is paramount to uncovering nascent patterns that provide further insight into the complexities of the human brain and distinguishing impairments.

Literature has demonstrated that conditions like hypoxia, Alzheimer’s, epilepsy, and other neurological issues cause neuronal impairments that change firing patterns. Modification of firing can potentially occur at the intracellular level of an individual neuron or at the intercellular level in how neurons propagate information to each other (neuronal interactions). However, the non-linearity of the processes at both the intracellular and intercellular level are caused by dynamic behavior^7^, making it difficult to capture explanatory responses. At the intracellular level, neuronal firing, or the generation of an action potential, demonstrates non-linearities in how thresholding and saturation phenomenon are governed^7^. At the intercellular level, neuronal interactions occur spatially giving a second dimension to these non-linearities^7^. These combined neuronal interactions are summed, potentially enabling subsequent neurons to meet their threshold criteria and thus fire as well^8,9^. These dynamic behaviors that demonstrate changes in threshold criteria and signal propagation can be modeled mathematically^9,10^. However, if a neuron is impaired, it has the potential to impede transmission and prevent subsequent neurons from firing, causing neurons to be functionally and electro-physiologically isolated^11^. A single, simulated EEG oscillation that would be produced by functional and impaired networks of neurons has been created to provide insight into the development of the novel methods presented in this paper.

### Prior work

EEG signals are characterized as non-linear time series because of complexities in cellular processes and signal propagation^7,11,12^. Though these complexities in neuronal firing occur even in the case of simple cognitive changes (e.g., sleeping), signal propagation is altered, which ultimately affects whether a neuron’s threshold criteria is met or not^13^. Collectively, changes in neuronal signal propagation affect global measurements of electrical activity as measured by the changing intensity/power of EEG band-limited waveforms (e.g., alpha, delta, theta). Based on this rudimentary and fundamental point, this relationship between cognitive states and EEG patterns was first documented by Berger^14^, who noted an attenuation in alpha waves (8–12 Hz) when comparing conscious waking states with rest/sleep^15^.

These observations became visibly apparent because the EEG alpha frequency is band-limited (8–12 Hz) and its intensity is more dominant during specific conditions (such as sleep). Thus, spectral intensity analysis methods have been the hallmark approaches for EEG analysis^7,12^, and Fourier methods have been the typical method for analyzing the intensity of specific frequency bands (i.e., delta *δ*, theta *θ*, alpha *α*)^16,17^. However, EEG signals are non-stationary^12^, making Fourier approaches problematic since they assume that the signal is infinitely long and stationary^18^. We are required to make this assumption about the signal being analyzed because Fourier uses sine and cosine waves as its basis function in order to decompose the signal into its appropriate frequency components. To overcome the issue of non-stationarity, Short-Time Fourier Analysis (STFT) is applied^17^. STFT permits the assumption of signal stationarity by applying windowing (typically into segments of 5–30 second windows)^19^. This type of analysis prevents one from examining how and when frequencies and intensities change within a windowed time segment. This dilemma has been previously identified as it relates to physiological signals^18^ and is known as the signal processing uncertainty principle^20^, which is related to Heisenberg’s uncertainty principle in physics^21^. This leads to the Heisenberg uncertainty principle’s fundamental trade-offs related to signal processing^18^: In order to obtain an increased time resolution, one loses frequency resolution. Likewise, in order to gain better frequency resolution, one loses time resolution^18^. Furthermore, strictly examining the raw intensity leads to high variability between subjects because variability is inevitably imposed by electrode conductance. Additional methodologies are required to combat this issue.

More current literature aims to determine more suitable non-linear analyses for examining EEG complexity using various types of entropy measurements to obtain informative features that can detect cognitive states and diseases^2,7,12,22^. The general underlying concept of these entropy measurements is based on examining complex sequences for similarities in patterns to quantify the predictability of the sequence. If the entropy is low, there are many patterns that are similar and the sequence is highly predictable. On the other hand, if the entropy is high, the sequence has fewer similar patterns and is less predictable.

There are numerous ways one can quantify similarity, and hence, various methods for calculating entropy^23^. Sleigh and Abasolo both discuss two possible families of entropy estimators with regard to EEG entropy signal analysis^7,24^. The first family consists of “phase-space embedding entropies”, which are designed to estimate the signal in the time domain. Popular methods within this family consist of approximate, Shannon, phase, sample, Kolmogorov, fuzzy, and permutation entropy^2^. As depicted in Table 1, these frequency-specific EEG waveforms (e.g., alpha, gamma) have been shown to indicate certain cognitive states and to have contextual meaning associated with brain damage and disease based on decades of supporting literature^25–27^. Even high gamma frequencies are now being related to motor and cognitive tasks^28^. However, this “phase-space” family of entropy methods does not examine similarity with regard to the frequency content of the signal. Thus, these temporal entropy approaches overlook a large part of the classical concepts of EEG analysis.

**Table 1:** EEG Waveform Interpretation.

EEG Waveform	Frequency	Interpretation
Delta	0-3.5 Hz	Sleep
Theta	4-7 Hz	Idling, Inhibition
Alpha	8-12 Hz	Relaxed/Reflecting
Low Beta	13-15 Hz	Slight Concentration
Mid Beta	15-18 Hz	Active Thinking
High Beta	18-40 Hz	Over-Arousal
Gamma	32-100 Hz	Sensory & Motor & Cognitive

The second family of entropy estimators is referred to as “spectral entropy” methods, which include spectral and Normalized Bispectrum Entropy methods. These methods aim to examine entropy from a frequency perspective, but at the cost of losing temporal information due to spectral windowing limitations (i.e., STFT). Furthermore, these methods typically utilize Fourier analysis methods which, as stated above, are inappropriate for EEG analysis due to the stationarity assumptions Fourier analysis imposes and the lack of granularity in changes signal frequencies and intensities^18^. Furthermore, these methods typically utilize Fourier analysis methods which, as stated above, are inappropriate for EEG analysis due to the assumption of stationarity imposed by Fourier analysis and the inability to detect granular changes in signal frequencies and intensities^18^.

From an analytical standpoint, none of these entropy methods used to characterize the non-linearities of EEG signals capture the intensity of specific EEG waveform spectral properties continuously over time, nor do they attempt to calculate precise dynamic temporal changes. Thus, there is no complexity method that can explain both the temporal and spectral complexity relationships. An analysis method that identifies intensity changes over time would provide a new understanding of the non-linear dynamics present in EEG signals.

### Motivation

**Figure 1:**
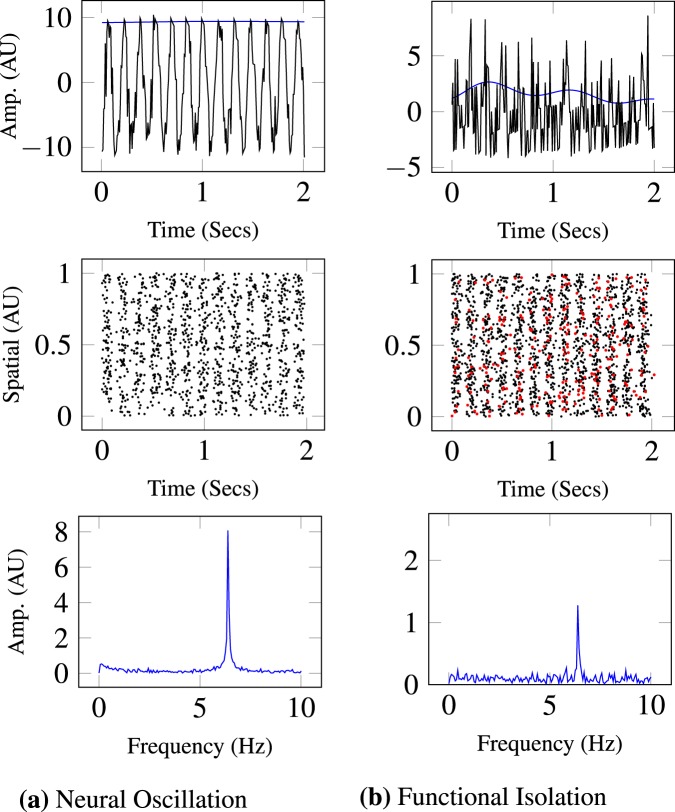
A rudimentary depiction of a standard neural oscillation in column 1 and a neural oscillation where functional neuronal isolation occurs, altering the local field potential in column 2. These two local field potentials in black are demonstrated in columns 1 and 2 in the first row. The characterized intensity of the signal over time is in blue. The second row of figures are the simulated spikes of individual neurons, where each dot represents an action potential in space and time. The red dots in the second column are the neurons that were suppressed and did not fire. The third row is the power spectrum using the Fourier transform of the local field potential neuronal oscillation. Intensity of the waveforms in the time domain and frequency domain are both highlighted in blue in rows 1 and 3.

From a physiological standpoint, the complex neuronal dynamics resulting from a method that would measure both temporal and spectral complexity relationships in firing patterns could potentially provide new information. The notion of complex neuronal networks, which generate these fundamental neuronal oscillations, has been backed by a vast amount of literature^8,15,29^. In order to capture the complexity of these dynamics, we propose the use of a rudimentary example with a simulated EEG that examines a simplistic network to enable the development of these methods (see Fig. 1). Figure 1 presents two cases: column one depicts a standard band-limited neuronal oscillation; and column two depicts a band-limited neuronal oscillation where a subset of neurons are functionally isolated (i.e., a neuron is impaired and has the potential to impede transmission and prevent subsequent neurons from firing). As previously discussed, we can observe these EEG oscillatory patterns through global field potentials at localized recording sites on the scalp, which are generated by the summation of large populations of neuronal action potentials. These populations of synchronized neuronal action potentials (in black) are shown in Fig. 1 in row two. In column two, the action potentials that did not fire are shown in red. These action potentials are summated at the macroscopic level and are viewed at the level of global field potentials shown in Fig. 1, row 1, in black, with the intensity in blue. It is worth noting that the intensity is attenuated via the analysis of the power spectrum (in row 3) and the characterized intensity over time, both in blue.

Moreover, the chaotic nature of the intensity over time is increased. Currently, the non-linear dynamics of how and when these intensities are altered over time are not captured with these methods (seen in blue in Fig. 1 of the first row). Note how a band-limited frequency and its intensity can change over time and become more unpredictable. The temporal intensity dynamics can potentially be altered at higher rates, where the ranks of the different spectral bands can alter in dominance. Outside this simulation, higher rates of change within temporal dynamics during hypoxia-ischemia has been reported in sheep^30^ and epilepsy^31^. However, current entropy measurement windowing techniques do not pinpoint instantaneous changes with regard to intensity, frequency, and time. This limitation calls for development of techniques capable of assessing whether there is additional information that could provide explanatory responses induced by neurological impairments (e.g., stroke, cancer).

Therefore, this work raises four relevant research questions: (1) Can we specifically measure EEG spectral waveforms (e.g., alpha) continuously over time to better capture changes in events? (2) How does the complexity of the intensity change for specific EEG spectral waveforms over time? (3) How do the proposed EEG entropy signal analysis methods compare to other standard measurements?

### Challenges

In order to detect instantaneous changes in intensity and relate them to the specified band-limited EEG waveforms in Table 1, a unique signal processing method must be developed. This method would share similarities to the DDWT method in order to capture intensity continuously over time. However, each filter would have to strictly capture the specified EEG waveforms and the adjacent filters would have to be considered in the method design to prevent redundant analysis of frequency content. If there is no redundancy, full reconstruction of the original signal can be achieved (plateau value of zero). This would mean that each filter is properly capturing only its specific intended EEG waveform. This requires intensive optimization since adjacent filter designs have dependencies on each other and are constrained by their cut-off frequencies with regard to specific EEG waveform bands. The second major consideration is how to formally apply these entropy measurements to the proposed signal processing methodologies. Although the wavelet entropy method analyzes the signal in a multi-resolution approach, it does not analyze instantaneous changes in the signal (granular time resolution). Instead, it examines entropy within the windowed time segment as a normalized sum of energy across the entire window. Thus, the resolution of any changes in the signal are generalized to the size of the window and do not examine these instantaneous changes in signals, which can inform granular, complex changes in firing. Finally, one must consider how to demonstrate that the new proposed approaches provide significant explanatory features when capturing these minuscule granular intensity changes from global field potentials.

### Insights

First, we test the feasibility of the proposed EEG algorithms using hypoxia data. Hypoxia is a state in which the body is unable to provide adequate levels of oxygen to its tissue. When oxygen levels are adequate, proper signal propagation between neurons can occur^11,32^. However, when oxygen deprivation occurs, the energy substrate supplied for neurons, Adenosine Triphosphate (ATP), is depleted, preventing synaptic transmission to other neurons^11,33^. The reduction of oxygen to tissue leads to neuronal electrophysiological isolation because of the inability to continue signal propagation^33,34^, thus altering the global measurements of the EEG recordings^11,34^.

Von Tscharner developed a signal processing intensity analysis method designed for Electromyography (EMG) signals using a wavelet-based analysis^35^. The design of these filters not only overcomes these time-frequency trade-offs, but the filters were designed in the frequency domain in order to minimize the plateau value of the filters to fully reconstruct the EEG signal^35^. The filter’s center frequencies and bandwidths were not chosen to capture any specific frequency ranges but designed to minimize the plateau value of the filter bank. Using optimization methods and these core concepts that von Tscharner presents, we can argue that the current design to fit appropriate optimal bandwidths specifically for continuous EEG analysis.

### Contributions

We propose a filter bank approach that addresses both the aforementioned challenges by examining the intensity of band-limited frequencies relevant to EEG in continuous time. Utilizing this developed intensity approach allows one to analyze entropy as a function of both time and frequency, unlike any current method available. We coin the term “EEG Activation Complexity” to refer to the calculation of entropy as the timing between a frequency band’s peak intensities. The contributions of this paper are:

1. Utilizing synthetic stationary and non-stationary signals, we capture a one-to-one mapping of intensity for the designed filters as a function of time.
2. We demonstrate that the timing of intensity peaks over a band-limited frequency is significantly less complex during normal oxygen conditions as compared to hypoxia conditions.
3. We demonstrate that the proposed method provide more information and add another dimension to the analysis of EEG signal processing.
4. We demonstrate activation complexity is a stronger predictor of cognitive impairment.

## Method

The methods section is partitioned into four sections. The first and second sections discuss the data, filter design, and optimization methods applied to produce the time-frequency intensity analysis. The third and fourth sections describe how we apply entropy calculations to the proposed time-frequency intensity analysis methods, where we introduce the EEG Activation Complexity.

### Hypoxia data set

The dataset was collected by a research team at NASA Langley Research Center (LaRC), who subjected 49 volunteers with current hypoxia training certificates to normobaric hypoxia to study the impact of hypoxia on aircraft pilot performance^36,37^. All participants consented to take part of the study as approved by the Institutional Review Board of NASA LaRC.

**Figure 2:**
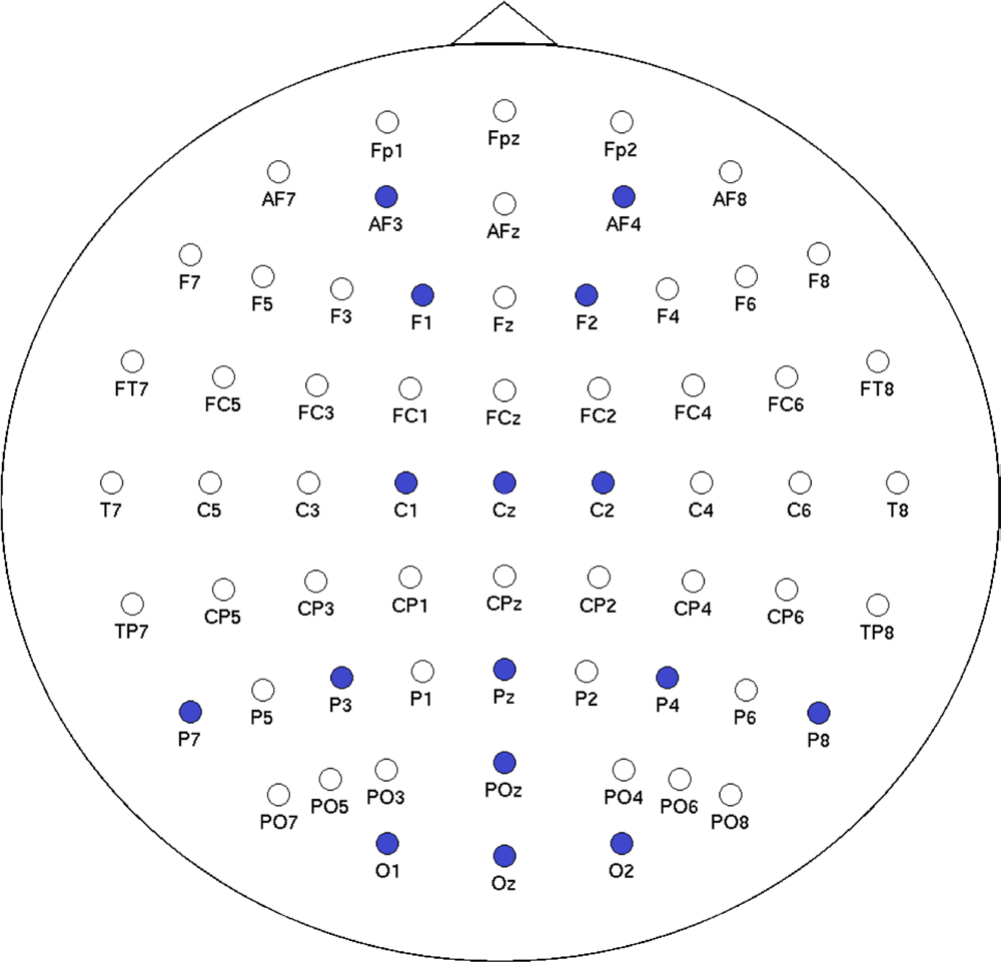
The electrode placement for the study is highlighted in blue.

The goal of the study was to understand cognitive impairment resulting from exposure to mild hypoxia in order to develop and test psychophysiologically-based adaptive automation/autonomous systems. Subjects in the study experienced simulated altitudes of sea level (21% O2) and 15,000 feet (11.2% O2) induced by an Environics, Inc. Reduced Oxygen Breathing Device (ROBD-2). During non-hypoxic (i.e., sea level) and hypoxic exposures, each subject experienced three 10-minute bouts performing three different tasks consisting of a battery of written tests, Multiple Attribute Task Battery (MATB)^38^, and flight simulation tasks. In each exposure, the research team collected task performance measures, a subjective self-reported workload (NASA Task Load Index TLX)^39^, and multiple physiological responses (including EEG). This article discusses only the EEG data collected during hypoxic and non-hypoxic exposures for the MATB, where the electrode configuration is provided in Fig. 2. As literature historically has shown, hypoxia induces cognitive performance deficits and changes within the EEG^11,30^. This has shown within this dataset’s past analyzes, were we demonstrated that during the hypoxic phase of the experiment subjects experienced statistically higher levels of perceived workload difficulty (NASA TLX)^36^, decreases to task performance (MATB)^40^, and changes in EEG power^37^. The specified electrode placement was used to avoid complications with the aviator’s oxygen mask component of the ROBD-2 breathing device which was worn using straps around the subject’s head.

### Filter bank intensity method

The goal of the filter bank design discussed in this paper is to develop a time-frequency intensity analysis for EEG-specific frequency bands. The general underlying concept of the filter design, which was motivated by von Tscharner^35^, is to extract the intensity of the signal as a function of time. In the proposed method, the filter bank comprises a collection of filters which, when summed, result in a relatively low plateau value across a range of frequencies (i.e., no one particular frequency dominates over any other).

#### Basis Function Definition

The spectral topology of a filter bank’s basis function is important because adjacent filters in the frequency domain must be summed to obtain a reasonably stable plateau value. Von Tscharner implemented a derivative of the Paul wavelet, where the filter bank design had arbitrary cutoff frequencies for the filters. Von Tscharner’s design was acceptable for analysis of EMG signals since it was only concerned with covering the entire range of possible frequencies and maintaining a low plateau value. However, this design choice is not suitable for our application, where individual EEG bands need to be extracted. Additionally, we found that the Paul wavelet could not handle the additional constraints required to capture the specific EEG waveform frequencies defined in Table 1. We adopt a pragmatic approach in this paper whereby optimization routines are used to find filter parameters that produce a reasonably optimal plateau value, EEG cutoff frequencies, and avoid the time-consuming process of manually adjusting filter bank parameters for each filter bank component.

For our filter bank, we selected the “flattened” Gaussian^41^ basis function topology to balance the extraction of frequency bands of the EEG spectrum while maintaining an acceptable filter bank plateau value. In the filter bank, the *i*^*th*^ filter (*i* = 1, …, *K*, where *K* is the total number of filters) within the frequency space is defined as

1 $${\widehat{\psi }}_{i}(f;f{c}_{i},{a}_{i},{b}_{i})={e}^{-{a}_{i}{(f-f{c}_{i})}^{2}-{b}_{i}{(f-f{c}_{i})}^{4}}\cdot \Theta (f)$$

which is parameterized by the center frequency *fc*_*i*_, and the tuning parameters *a*_*i*_, and *b*_*i*_. The Heaviside function, *Θ*(*f*), constrains the design to only positive frequencies (*f* ≥ 0). The filter bank is constructed using non-linear scaling to the basis function ($\widehat{\psi }$) in the time domain by shifting each center frequency and tuning the parameters to achieve a better filter bank design. Since these tuning parameters are not constant and altered for each *i*^*th*^ filter, we refrain from referring to this design as a wavelet implementation and instead refer to it simply as a filter bank design. However, these filters maintain the same generalized basis function (a “flattened” Gaussian) and the core concepts of design followed by von Tscharner^35^ while still fulfilling a wavelet’s admissibility criterion.

#### Filter bank optimization

The ultimate goal for our filter bank design was to find a set of filters with an acceptable (near optimal) plateau value. We attempted fitting filter parameters *fc*_*i*_, *a*_*i*_, and *b*_*i*_ by hand, but acceptable results were time-consuming to achieve. We also tried optimizing the entire filter bank, but the results were not acceptable and were found to be computationally complex. We modified our approach to optimize sets of three filters at a time. We were able to achieve better results in less time with this approach, but we encountered difficulties in seeding the optimization routines with reasonable center frequencies. We attempted to solve this problem by optimizing only the placement of center frequencies such that the center frequencies were uniformly spaced with considerations for the boundaries imposed by EEG band cutoffs. We then used these center frequencies to seed the optimization of sets of three filters. After iterating through all filters in the filter bank, slight manual adjustments to the parameters *a*_*i*_ and *b*_*i*_ were made to better adjust for the plateau value.

We introduced the following generalized optimization approach for developing the proposed filter bank for the constraints of this particular EEG. A general overview of the method is as follows:

Step 1: Select a number of wavelets to represent each band of the EEG spectrum and estimate the spacing of wavelet center frequencies by minimizing the sum of differences between separations of adjacent wavelet center frequencies.
Step 2: Use the set of center frequencies from Step 1 to approximate the optimal plateau value for the entire wavelet filter bank by optimizing sets of three wavelets.

The details of each of these steps is outlined in the following sections.

*Step 1: Estimated Optimal Spacing*

The goal of the Step 1 is to find a set of center frequencies (*fc* ∈ **R**^*K*^, where *K* is the total number of wavelets selected) to seed the optimization routine in Step 2. This set is found by minimizing the sum of differences between separations of adjacent wavelet center frequencies as defined in the following optimization problem

$$\mathop{{\rm{minimize}}}\limits_{fc}\,\mathop{\sum }\limits_{i=1}^{K-2}{((f{c}_{i+1}-f{c}_{i})-(f{c}_{i+2}-f{c}_{i+1}))}^{2}$$

$$\mathrm{subject}\,\mathrm{to}\,f{c}_{i}^{lb}\le f{c}_{i}\le f{c}_{i}^{ub},\ i=1,\ldots ,K,$$

where $f{c}_{i}^{lb}$ and $f{c}_{i}^{ub}$ are the lower and upper constraint boundaries on the center frequencies. These boundaries can be considered either “hard” – set by the halfway point between the minimum lower and upper boundaries of the EEG band and the halfway point between the maximum lower and upper boundaries of the EEG band (as found in 1), or “soft” – given by the approximate acceptable regions of center frequencies that are not fully determined by the EEG band ranges in Table 1.

**Figure 3:**
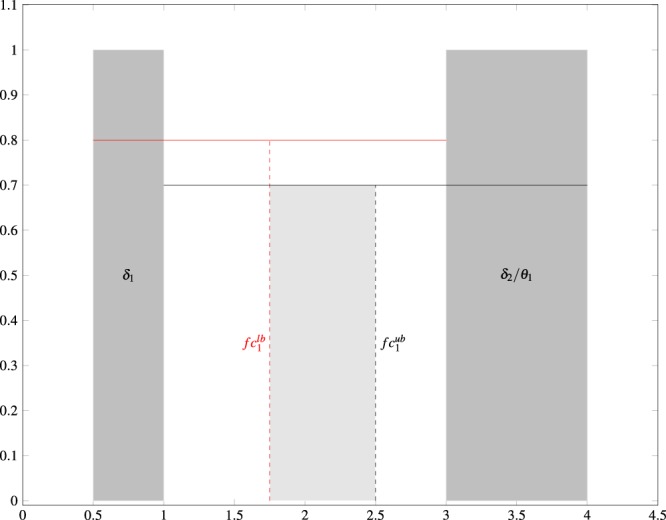
Figure depicting the calculation of the center frequency’s acceptable range for the *δ* EEG band (i.e., $f{c}_{\delta }^{lb},f{c}_{\delta }^{ub}$).

An example of a “hard” boundary exists for a single wavelet occupying the *δ* band (*fc*_1_; starting between 0.5 Hz to 1 Hz and ending between 3 Hz to 4 Hz; see Fig. 3). This constraint is considered “hard” because it is imposed by the adjacent *θ* band to the right and the undefined region of negative frequency to the left. This wavelet should have a center frequency that is between 1.75 Hz ($f{c}_{1}^{lb}$; halfway between 0.5 Hz to 3 Hz) and 2.5 Hz ($f{c}_{1}^{ub}$; halfway between 1 Hz to 4 Hz; see Fig. 3).

**Figure 4:**
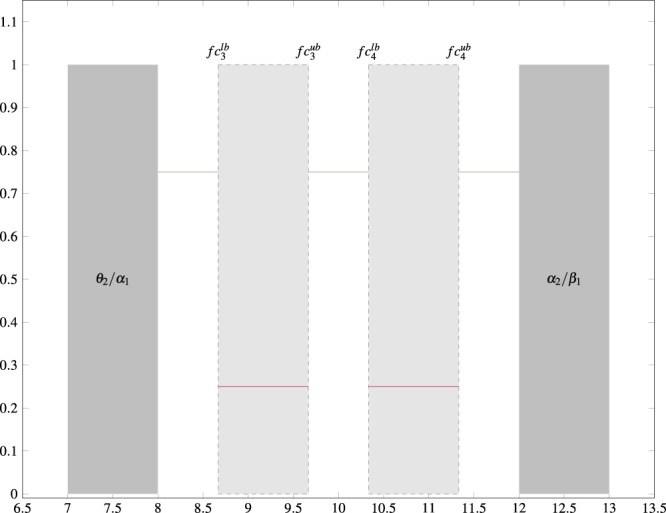
Figure depicting the calculation of multiple center frequency acceptable ranges for the *α* EEG band (i.e., $f{c}_{\alpha }^{lb},f{c}_{\alpha }^{ub}$).

An example of a “soft” boundary exists for two wavelets occupying the *α* EEG band, which contains frequencies starting between 7 Hz to 8 Hz and ending between 12 Hz to 13 Hz (see Fig. 4). The constraints on these two center frequencies are considered “soft” because the single EEG band (*α* in this case) is represented by two wavelets, and at least one of the boundaries for each wavelet is “artificially” imposed. For the case of the first filter in the *α* band, the *θ* EEG band is to the left (ending between 7 Hz to 8 Hz) for the first wavelet, but the upper bound is not well-defined since we have some choice as to the next *α* band filter.

These constraints help ensure that the cutoff frequency (*fco*_*i*_) of each filter would take the value of 1/*e* (i.e., ${\widehat{\psi }}_{i}(f=fc{o}_{i})=1/e$) between the frequency range specified by the boundaries of certain EEG bands during Step 2. Furthermore, center frequencies that are spaced evenly were found to produce more stable plateau values in Step 2. The aforementioned optimization problem was solved using the non-linear constrained optimization routine (“fmincon”) in MATLAB.

*Step 2: Optimize Basis Function Parameter Values* The goal of Step 2 is to produce a filter bank that equally represents all EEG frequencies within the range of interest while providing reasonable separability of different EEG bands as defined in the scientific literature (see Table 1). One possible way to achieve such a filter bank is to find the set of filter parameters that minimize the path length integral of the sum of all filters in the filter bank. Colloquially, this amounts to finding the shortest distance between two points (a straight line in Euclidean geometry). Any deviations from a straight line result in having to “walk” a greater distance between the starting and ending frequencies. For an entire set of *K* filters, this objective can be operationalized as the path length integral between the first and last center frequencies, written as

2a $$\mathop{{\rm{minimize}}}\limits_{fc,a,b}\,{\int }_{f{c}_{1}}^{f{c}_{K}}dfL$$

2b $$\mathrm{subject}\,\mathrm{to}\,{f}_{j}^{lb}\le f{c}_{j}\le {f}_{j}^{ub}$$

2c $${a}_{j}^{lb}\le {a}_{j}\le {a}_{j}^{ub}$$

2d $${b}_{j}^{lb}\le {b}_{j}\le {b}_{j}^{ub}$$

2e $${f}_{j}^{L}\le fc{o}_{j}^{L}(f{c}_{j},{a}_{j},{b}_{j})\le {f}_{j}^{H}$$

2f $${f}_{j+1}^{L}\le fc{o}_{j}^{U}(f{c}_{j},{a}_{j},{b}_{j})\le {f}_{j+1}^{H}$$

2g $$\widehat{\psi }\left(f=f{c}_{j-1};f{c}_{j},{a}_{j},{b}_{j}\right)\le \epsilon $$

2h $$\widehat{\psi }\left(f=f{c}_{j+1};f{c}_{j},{a}_{j},{b}_{j}\right)\le \epsilon $$

2i $$\mathrm{where}\,j=1,\ldots ,K$$

where *fc*, *a*, *b* ∈ **R**^*K*^ and *L* is the arc length of the sum of all filters between the center frequency of the first and last filter in frequency space

3 $$L(f;fc,a,b)=\sqrt{1+{\left(\frac{d}{df}\left(\mathop{\sum }\limits_{i=1}^{K}{\widehat{\psi }}_{i}(f;f{c}_{i},{a}_{i},{b}_{i})\right)\right)}^{2}}.$$

As in Step 1, the possible ranges of center frequencies are constrained (Eq. 2b) as well as the parameters *a* and *b* (Eqs. 2c and 2d). Additionally, Eq. 2e and Eq. 2f ensure that the lower and upper cutoff frequencies $fc{o}_{j}^{L}(f{c}_{j},{a}_{j},{b}_{j})$ and $fc{o}_{j}^{U}(f{c}_{j},{a}_{j},{b}_{j})$ for the *j*^*th*^ filter given by

4 $$fc{o}_{j}^{L}(f{c}_{j},{a}_{j},{b}_{j})=f{c}_{j}-\sqrt{\frac{\sqrt{{a}_{j}^{2}+4{b}_{j}}-{a}_{j}}{2{b}_{j}}},$$

and

5 $$fc{o}_{j}^{U}(f{c}_{j},{a}_{j},{b}_{j})=f{c}_{j}+\sqrt{\frac{\sqrt{{a}_{j}^{2}+4{b}_{j}}-{a}_{j}}{2{b}_{j}}}$$

fall within the acceptable ranges for the associated EEG band. Finally, in order to produce a reasonably biorthogonal filter bank, the value of the *j*^*th*^ filter at the center frequencies of the (*j*−1)^*th*^ and (*j*+1)^*st*^ filters are constrained to be less than or equal to *ϵ* = 0.0005 through constraints Eqs. 2g and 2h.

**Figure 5:**
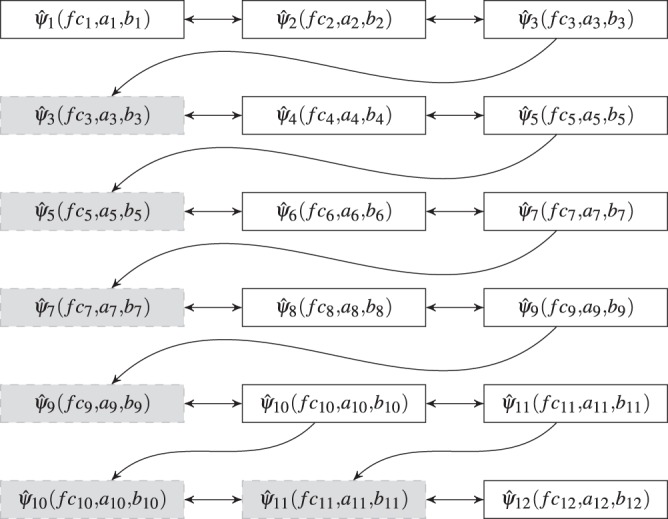
Diagram showing filter parameters optimized during each round of Step 2. Each row corresponds to an optimization round. Boxes shaded gray represent filters whose parameters were fixed during an optimization round.

Unfortunately, our attempts at directly optimizing Eq. 2a were met with poor results. However, we were able to approximate the global optimum by sequentially considering only three filters at a time until all *K* filters’ parameters were determined (see Fig. 5). As such, Eq. 2a was modified to account for three filters (${\widehat{\psi }}_{i}$, ${\widehat{\psi }}_{i+1}$, and ${\widehat{\psi }}_{i+2}$) at a time as described in the following optimization problem

6a $$\mathop{{\rm{minimize}}}\limits_{fc,a,b}\ \ \ \ \ \frac{{\int }_{f{c}_{i}}^{f{c}_{i+2}}df\left[L-(f{c}_{i+2}-f{c}_{i})\right]}{(f{c}_{i+2}-f{c}_{i})}$$

6b $$\mathrm{subject}\,\mathrm{to}\,f{c}_{j}^{lb}\le f{c}_{j}\le f{c}_{j}^{ub}$$

6c $${a}_{j}^{lb}\le {a}_{j}\le {a}_{j}^{ub}$$

6d $${b}_{j}^{lb}\le {b}_{j}\le {b}_{j}^{ub}$$

6e $${f}_{j}^{L}\le fc{o}_{j}^{L}(f{c}_{j},{a}_{j},{b}_{j})\le {f}_{j}^{H}$$

6f $${f}_{j+1}^{L}\le fc{o}_{j}^{U}(f{c}_{j},{a}_{j},{b}_{j})\le {f}_{j+1}^{H}$$

6g $$\widehat{\psi }\left(f=f{c}_{j-1};f{c}_{j},{a}_{j},{b}_{j}\right)\le \epsilon $$

6h $$\widehat{\psi }\left(f=f{c}_{j+1};f{c}_{j},{a}_{j},{b}_{j}\right)\le \epsilon $$

6i $$\mathrm{where}\,j=i,i+1,i+2$$

where the arc length along the three consecutive filters *L* is given by

7 $$L(f;fc,a,b)=\sqrt{1+{\left(\frac{d}{df}({\widehat{\psi }}_{i}+{\widehat{\psi }}_{i+1}+{\widehat{\psi }}_{i+2})\right)}^{2}},$$

and the constraints given in Eq. 6b to Eq. 6h serve the same functions as with the optimization problem in Eq. 2a.

For the first three filters, *fc*_1_, *a*_1_, *b*_1_, *fc*_2_, *a*_2_, *b*_2_, *fc*_3_, *a*_3_, and *b*_3_ are found such that Eq. 6a is minimized. The optimal values of filter 3 are then used in the next round to find *fc*_4_, *a*_4_, *b*_4_, *fc*_5_, *a*_5_, *b*_5_ such that Eq. 6a is minimized. This process is repeated until all *K* filter parameters have been optimized (see Fig. 5).

As with Step 1, the method in Step 2 was implemented using the non-linear constrained optimization (fmincon) routine in MATLAB.

#### Optimized filter parameters

**Figure 6:**
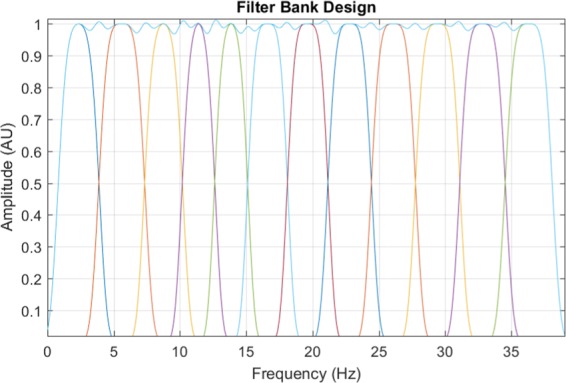
An optimized filter bank design defined by the parameters provided in Table 2.

Utilizing the proposed methodology in which the constraints of the filtering paradigm are accounted for and optimized, we obtain Table 2. These parameters are then applied to Eq. 8 and shown in Fig. 6, where we can note the plateau vector, **PV**(*f*), is defined as

8 $${\bf{PV}}(f)=\mathop{\sum }\limits_{i=1}^{K}{e}^{-{a}_{i}{(f-f{c}_{i})}^{2}-{b}_{i}{(f-f{c}_{i})}^{4}}\cdot \Theta (f),$$

where $\forall f\in \bar{1,S}$, *S* = *F*_*s*_∕2, and *F*_*s*_ is the sampling frequency. The plateau value, *P*_*v*_, is obtained by calculating the standard deviation of the vector **PV**.

**Table 2:** Filter Bank Parameters (*P*_*v*_ = 0.0091).

Filter *i*	*Cf*_*i*_ (Hz)	*a*_*i*_	*b*_*i*_	*Fp*_1_ (Hz)	*Fp*_2_ (Hz)
1	2.349	0.072	0.095	0.6	4.0
2	5.605	0.001	0.077	3.8	7.6
3	8.759	0.101	0.119	7.2	10.4
4	11.400	0.219	0.161	10.0	12.8
5	13.859	0.170	0.180	12.4	15.2
6	16.608	0.007	0.135	15.0	18.2
7	19.627	0.001	0.127	18.0	21.4
8	22.792	0.001	0.095	21.0	24.6
9	26.094	0.001	0.090	24.2	28.0
10	29.432	0.001	0.088	27.6	31.2
11	32.820	0.003	0.078	31.0	34.8
12	36.307	0.001	0.070	34.4	38.2

#### EEG Filter Implementation

**Figure 7:**
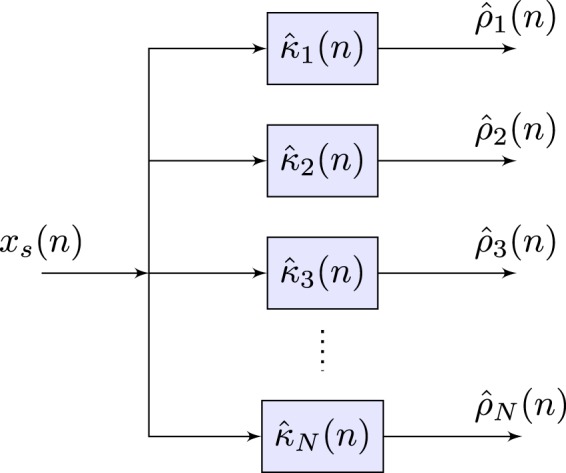
A Generalized Filter Bank design that mimics an equalizer in which the input signal, *x*_*s*_(*n*), is decomposed into its respective intensity components, ${\widehat{\rho }}_{i}(n)$, via the filtering paradigm, ${\widehat{\kappa }}_{i}$.

This discussed method deviates from Von Tscharner’s classical approach of the filter implementation because of the valid concerns presented by Gabriel^42^. This discussion in^42^, points out how applying the designed filters to the EEG’s source signal in the frequency domain, *X*_*s*_(*f*), is inappropriate since we are applying the Fourier transform to a non-stationary signal, thus defeating one of the major purposes of the novel signal processing approach. As Borg highlights^43^, Von Tscharner’s implementation shares similarities to a basic equalizer which decomposes the EEG time domain’s source signal, *x*_*s*_(*n*), into its associated intensity components, *ρ*_*i*_(*n*), with respect to each filtering process, *κ*_*i*_, shown in Fig. 7.

The presented filtering process utilizes the EEG signal in the time domain, *x*_*s*_(*n*), where we obtain a frequency band-limit intensity, *ρ*_*i*_(*n*), over time by applying convolution with the designed filters, ${\widehat{\psi }}_{i}(n)$ and Gaussian smoothing methods. We define this entire process as, ${\widehat{\kappa }}_{i}$, where *i* ∈ {1, …, *K*} filters.

**Figure 8:**
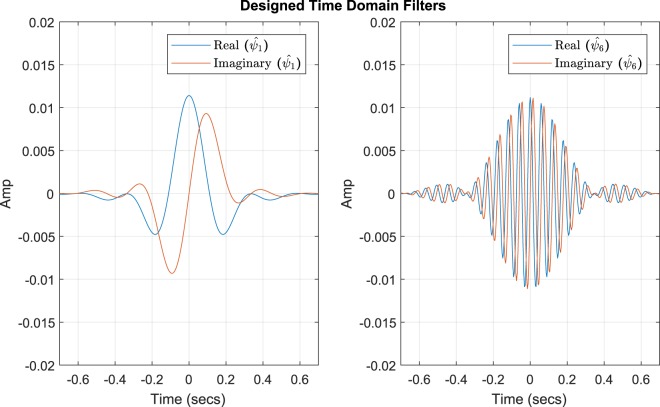
Visual representation ${\widehat{\psi }}_{i}(n)$ and ${\widehat{\psi }}_{i}(n)$ in the time domain.

In order to obtain ${\widehat{\psi }}_{i}(n)$, we transfer the designed respective frequency domain filter, ${\widehat{\psi }}_{i}(f{c}_{i},{a}_{i},{b}_{i})$, to the time domain by,

9 $${\widehat{\psi }}_{i}(n)={{\mathscr{C}}}^{{\mathscr{L}}}\{{{\mathscr{F}}}^{-1}\{{\widehat{\psi }}_{i}(f{c}_{i},{a}_{i},{b}_{i})\}\},$$

where ${{\mathscr{F}}}^{-1}$ is the inverse Fourier transform and ${{\mathscr{C}}}^{{\mathscr{L}}}$ is the circular shift of the numeric output of the function, where $L=\frac{N}{2}$ and *N* is the length of the filter in the time domain. The ${{\mathscr{C}}}^{{\mathscr{L}}}$ operation with $L=\frac{N}{2}$ is equivalent to performing a FFT shift, which adjusts the mirroring image in the frequency domain. However, this sequence happens to be in the time domain. Thus, the sequence {*x*(0), …, *x*(*N* − 1)} is cyclically shifted to {*x*(*N*∕2), *x*(*N* − 1), 0, …, *x*(*N*∕2 − 1)}. By applying Equation 9, we are able to move the filter designed in the frequency domain to the time domain described with real and imaginary components, shown in Fig. 8.

Utilizing the filter in time domain, ${\widehat{\psi }}_{i}(n)$, we obtain the intensity of the signal *x*_*s*_(*n*) by,

10 $${\rho }_{i}(n)=2\left|\mathop{\sum }\limits_{-\infty }^{\infty }{\widehat{\psi }}_{i}(n-m){x}_{s}(m)\right|,$$

11 $${\rho }_{i}(n)=2| {\widehat{\psi }}_{i}\ast {x}_{s},| $$

which is defined by the convolution of *x*_*s*_(*n*) with ${\widehat{\psi }}_{i}(n)$. The intensity sequence, *ρ*_*i*_(*n*), is then smoothed using a Gaussian filter,

12 $${G}_{f}(n)=\frac{1}{\sqrt{2\pi {\sigma }^{2}}}{e}^{-0.5{(\frac{x}{\sigma })}^{2}}$$

where $\sigma =\frac{{F}_{s}}{2}$, *F*_*s*_ is the sampling frequency and $x=\in \{\frac{-3{F}_{s}}{2},\ldots ,\frac{3{F}_{s}}{2}\}$. The intensity signal is convolved with the Gaussian filter to obtain the smoothed filtered EEG intensity:

13 $${\widehat{\rho }}_{i}(n)={\rho }_{i}(n)\ast {G}_{f}(n).$$

#### Activation complexity

**Figure 9:**
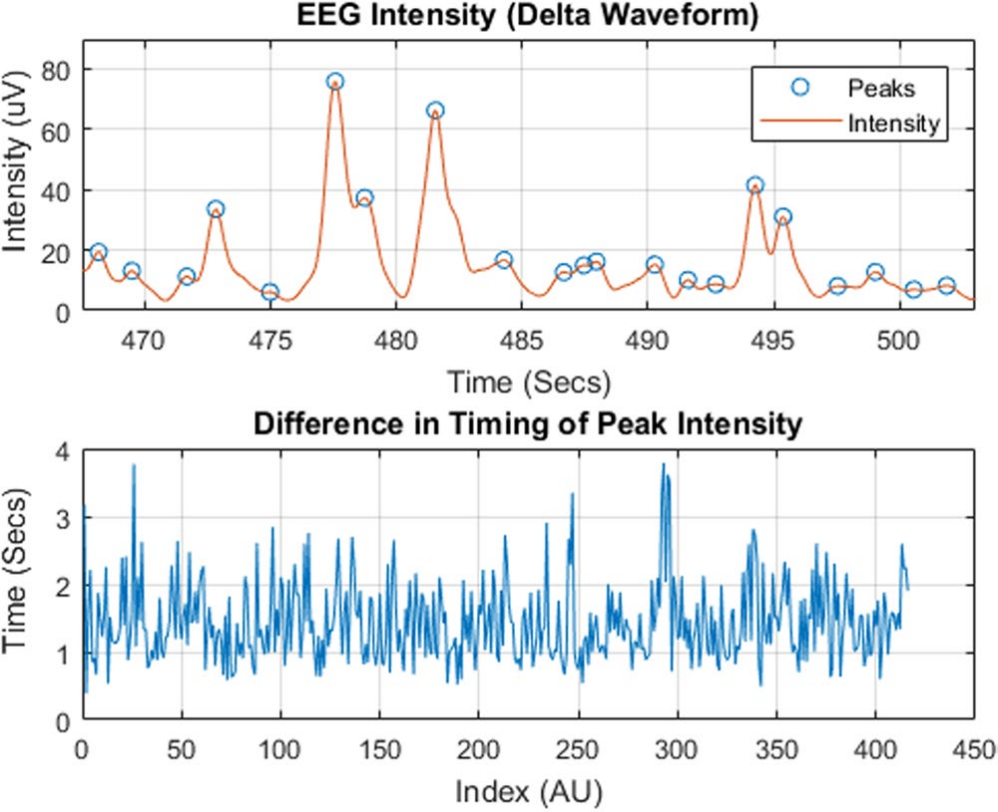
This figure represents the process for calculating Activation Complexity. The upper part of the figure shows the intensity of the designed delta waveform band, *ρ*_1_(*n*) in red, with the indicated peaks on the intensity band, *ρ*_1_(*n*) circled in blue. The differences in time between those peaks are calculated to form *ΔA*_*i*_(*k*), shown in the bottom image. A standard entropy measurement method can then be applied to this sequence.

We used Activation Complexity to examine the predictability of the intensity, ${\widehat{\rho }}_{i}(n)$, of specific EEG frequency ranges (e.g., *α*, *δ*) as a function of time. We calculate Activation Complexity using the proposed filter bank design, a peak detector, and a temporal entropy measurement (e.g., sample entropy). The peak detector implemented in this work utilized the function “findpeaks” from MATLAB version 2017a, where the function will produce a vector of indices for the locations in time where the peaks occur, *A*_*i*_(*k*). In the upper part of Fig. 9, we depict instances in time for the peaks of the intensity of the delta waveform frequencies. In the lower part of Fig. 9 is the vector *Δ***A**_1_, the sequence of the timing differences between all the peaks of the intensity waveform calculated simply by

14 $$\Delta {{\bf{A}}}_{i}=[{A}_{i}(2)-{A}_{i}(1),\ldots ,{A}_{i}(n)-{A}_{i}(n-1)].$$

Following this, we computed the sample entropy of the new sequence, Δ**A**_*i*_^23^ to produce the Activation Complexity measurement *Ac*_*i*_ for each EEG lead.

It is important to note that the type of entropy measurement applied to the sequence will be sensitive to the number of data points in the sequence, thus limiting the window size that can be analyzed. Typically, sample entropy and permutation entropy require a minimum of 100 samples, whereas approximate entropy requires a minimum of 1000 samples^23^.

## Evaluation and Discussion

In this section, we address the following research questions regarding the filter design intensity method and entropy approaches to distinguish changes in EEG firing patterns during hypoxic conditions at 15,000 feet of altitude versus non-hypoxic conditions at sea level:

1.     Can we accurately depict the intensity of the stationary and non-stationary signals proportional to the original time-series?
2.     Does the proposed Activation Complexity method demonstrate the ability to extract complexity EEG dynamic trends to distinguish brain activity under hypoxia?
3.     How do classical methods perform in distinguishing differences between hypoxia and sea level changes in the brain?
4.     How do classical spectral intensity compare to activation complexity for distinguishing cognitive impairment?

### RQ1: simulated continuous EEG intensity measurement

**Figure 10:**
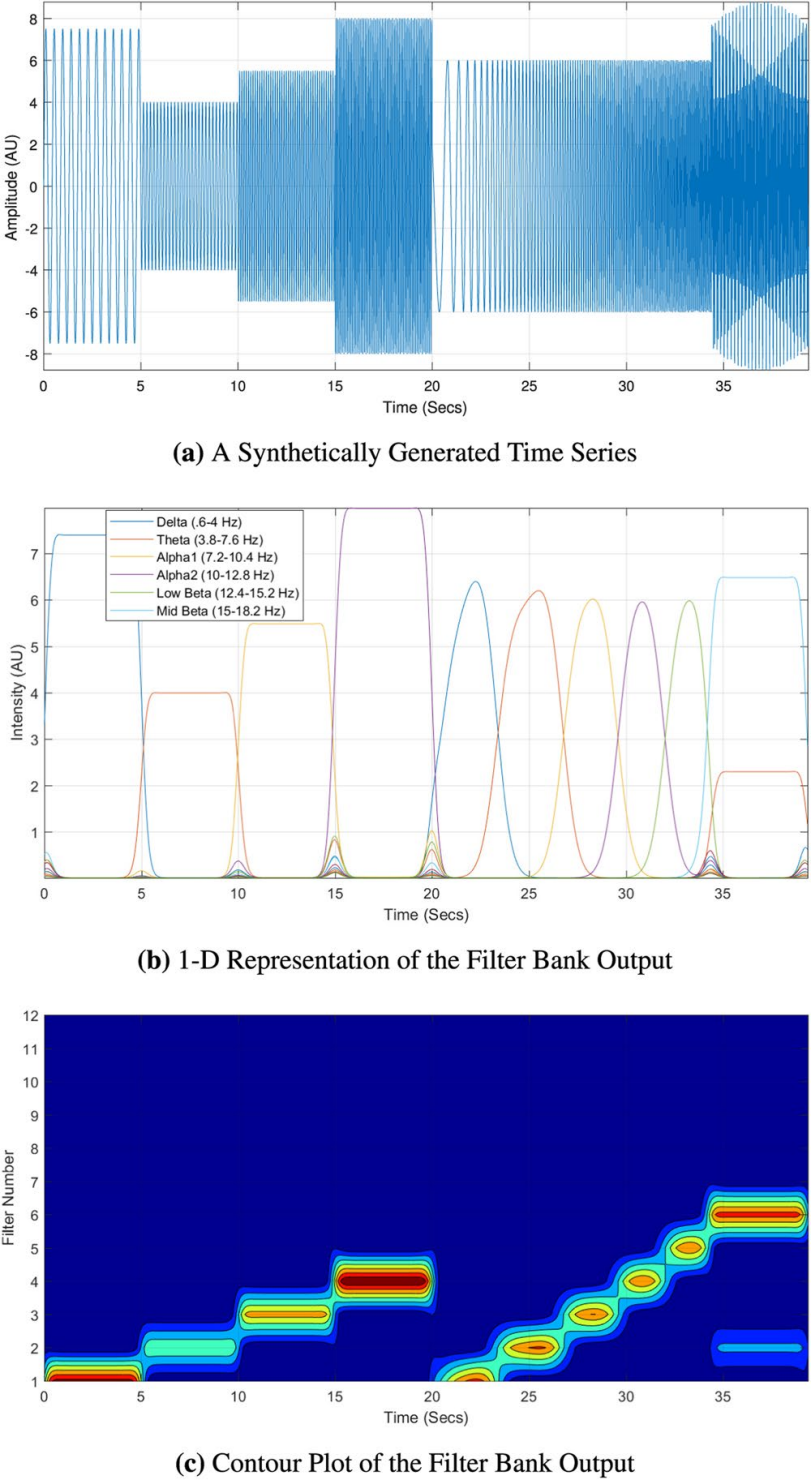
The synthetic time series consists of various stationary and non-stationary frequencies (a chirp from 20–30 seconds). We demonstrate two visual representations of the filter bank output. The 1-D representation allows for an enhanced comparison of an accurate depiction of intensity to the synthesized time series. The contour plot allows for a better global visualization of which filters are activated and their timing.

We hypothesize that by using the proposed filter bank methodology, we will be able to extract pertinent EEG frequency band intensity continuously over time. Utilizing the designed filter bank methodology, we simulate various stationary and non-stationary waveforms to evaluate intensity as a function of time. In Fig. 10a, we first modeled four stationary waveforms at frequencies of 2.3 Hz, 5.6 Hz, 8.75 Hz, and 11.4 Hz with amplitudes of 7.5, 4, 5.5, and 8, respectively. The fifth component is a non-stationary signal model using a chirp in which the frequency linearly increases from 0 to 15 Hz with an amplitude of 6. We then provide an example of a linear combination of two stationary signals at 2.3 Hz and 16.6 Hz with amplitudes of 2.3 and 6.5, respectively. All of these waveforms were concatenated together as a single time series. Thus, the transitions between waveforms were abrupt and discontinuous, causing mild perturbations in irrelevant filters to activate. Through visual inspection in Fig. 10, we can obtain an equivalent proportionality to the simulated waveform generated in Fig. 10a. Figure 10b can be represented in a two-dimension fashion, similar to how continuous wavelet transforms are presented using contour plots shown in Fig. 10c. This allows a clearer depiction of time, frequency (i.e., filter number), and intensity.

### RQ2: activation complexity

**Figure 11:**
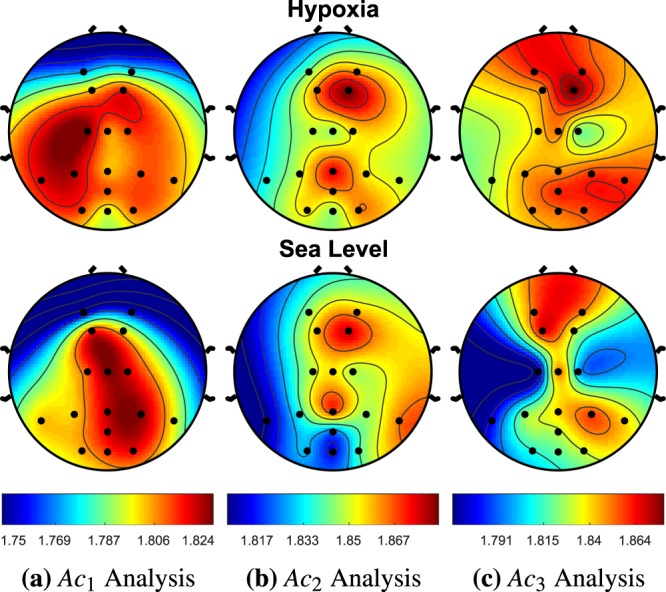
AC Analysis, *Ac*_*i*_, is shown between the hypoxia and sea level cohorts for the first three intensity filter designs (delta [0.6–4.0 Hz], theta [3.8–7.6 Hz], low alpha [7.2–10.4 Hz]), where i is the applied filter. *Ac*_1_ demonstrated no significant changes across any of the EEG sites. *Ac*_2_ demonstrated a significant increase in entropy (i.e., complexity) at the *PO*_*z*_ EEG site with a p-value of 0.033. *Ac*_3_ produced an increase in complexity at the *P*_7_, *F*_2_, and *C*_1_ with p-values of 0.046, 0.004, 0.0001, respectively.

**Figure 12:**
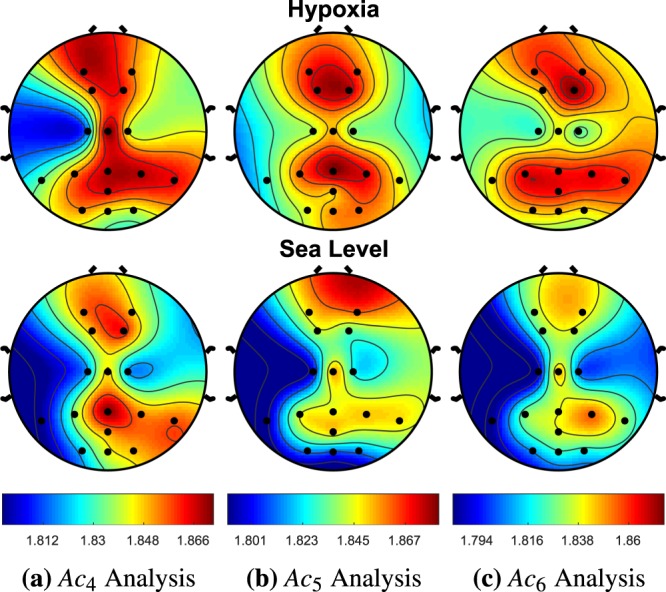
*Ac*_4_ demonstrated no significant changes across any of the EEG sites. For hypoxia, *Ac*_5_ demonstrated a significant increase entropy (i.e., complexity) at *P*_7_, *P*_*z*_, *O*_*z*_, *O*_2_, *F*_1_, *F*_2_, *C*_2_ with p-values of 0.049, 0.021, 0.006, 0.007, 0.033, 0.018, and 0.028, respectively. During hypoxia *Ac*_6_ produced a significant increase in complexity at the *P*_7_, *O*_1_, *F*_2_, *C*_1_, *P*_3_ with p-values of 0.0003, 0.049, 0.017, 0.0464, and 0.033, respectively. It is also worth noting that *O*_1_ had p-values of 0.065 and 0.060 for *Ac*_4_ and *Ac*_5_, respectively.

**Figure 13:**
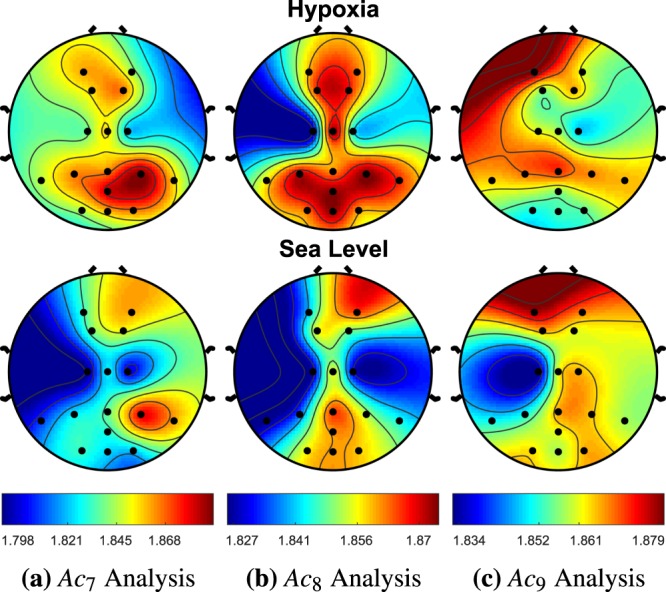
The hypoxia cohort for *Ac*_7_ produced an increase in complexity for EEG sites *P*_7_, *Po*_*z*_, *P*_*z*_, 0_2_, *C*_1_, *P*_3_ with p-values 0.036, 0.048, 0.026, 0.040, 0.045, 0.035, respectively. The hypoxia cohort for *Ac*_8_ produced an increase in complexity for EEG sites *P*_7_, and *P*_3_ with p-values 0.031 and 0.035, respectively. *Ac*_9_ demonstrated no significant changes across any of the EEG sites.

**Figure 14:**
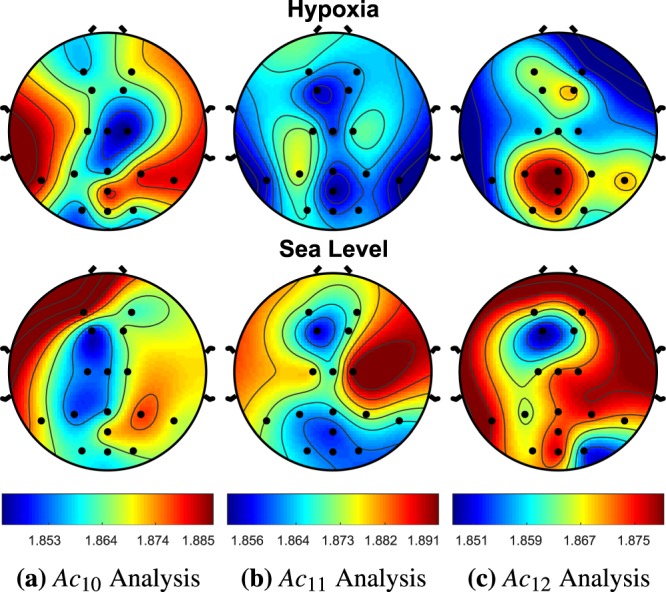
The hypoxia cohort for *Ac*_10_ produced a decrease in complexity for EEG sites *AF*_4_ with a p-value of 0.019. *Ac*_11_ also produced a significant decrease in complexity at *C*_2_ with a p-value of 0.047. *Ac*_12_ demonstrated no significant changes across any of the EEG sites.

Activation Complexity (AC) is applied to a hypoxia data set, where we hypothesize that this novel method can extract irregular neuronal firing patterns from global EEG recordings. This method was applied to EEG data collected from 49 subjects exposed to three 10 minute bouts of normobaric hypoxic (12% O2/15k ft) and non-hypoxic conditions (22% O2/sea-level) at NASA Langley Research Center. One-sample t-tests and bootstrapped t-tests for multiple comparisons were used between the two cohorts for the computerized MATB task bout of hypoxic and sea level conditions. The AC analysis using sample entropy used a template length of m = 2 and a threshold value of r = 0.25, producing 26 different statistically significant AC measurements across filters and EEG recording sites for when no multiple comparison correction (NMCC) as applied. When a bootstrap multiple comparison correction (MCC) was applied, 13 different statistically significant AC measurements across filters and EEG recording sites was demonstrated. The visual changes of AC between conditions are shown in Figs. 11, 12, 13 and 14. We explored various other threshold values of .15, .2, and .3, which produced 17, 19, and 25 statistically different activation complexities, respectively (For the NMCC case). The AC measurements that demonstrated significant changes utilizing the other threshold values demonstrated similar patterns with regard to EEG leads and frequency bands. The calculated AC complexity was normalized across all EEG leads, incorporating both the hypoxia and sea level cohorts associated with each filter bank’s intensity analysis. This was done to highlight the differences in complexity, as depicted in the colored contour map in Figs. 11, 12, 13 and 14. The details of the results regarding EEG site location and p-value for the one-sample test (for *α* ≤ 0.05 with *N* = 47) are provided in the comments below the figure. Additionally, Table 3, is provided for more details regarding the means and standard errors for instances of *α* ≤ 0.1.

**Table 3:** Activation Complexity.

EEG	Hypoxia		Sea Level		p-value	p-value
Site	*Mean*_*H*_	*SE*_*H*_	*Mean*_*SL*_	*SE*_*SL*_	NMCC	MCC
*Ac*_2_: *Frequency* *Range* (3.8–7.6 Hz)						
*P*0_*z*_	1.87	0.011	1.832	0.013	0.034	0.048
*Ac*_3_: *Frequency* *Range* (7.2–10.4)						
*P*_7_	1.844	0.018	1.801	0.025	0.046	x
*O*_1_	1.861	0.017	1.830	0.020	0.061	x
*O*_*z*_	1.865	0.012	1.836	0.013	0.083	x
*F*_2_	1.888	0.010	1.848	0.009	0.004	0.004
*C*_1_	1.855	0.020	1.791	0.025	0.0001	0.047
*Ac*_4_: *Frequency* *Range* (10.0–12.8)						
*P*_7_	1.846	0.0154	1.812	0.024	0.065	x
*C*_*z*_	1.885	0.0089	1.860	0.013	0.098	x
*O*_1_	1.864	0.012	1.836	0.017	0.064	x
*Ac*_5_: *Frequency* *Range* (12.4–15.2)						
*P*_7_	1.837	0.018	1.801	0.024	0.049	x
*P*_*z*_	1.890	0.008	1.855	0.013	0.021	0.028
*O*_1_	1.854	0.015	1.823	0.020	0.060	x
*O*_*z*_	1.865	0.012	1.818	0.019	0.006	0.035
*O*_2_	1.868	0.009	1.823	0.016	0.007	0.017
*F*_1_	1.883	0.011	1.851	0.010	0.033	0.034
*F*_2_	1.884	0.010	1.842	0.012	0.018	0.008
*C*_1_	1.850	0.020	1.809	0.028	0.028	x
*Ac*_6_: *Frequency* *Range* (15.0–18.2)						
*P*_7_	1.849	0.016	1.794	0.022	0.0003	0.045
*P*_8_	1.867	0.014	1.839	0.017	0.084	x
*O*_1_	1.854	0.018	1.818	0.021	0.049	x
*F*_2_	1.881	0.011	1.844	0.012	0.017	0.023
*C*_1_	1.832	0.023	1.798	0.030	0.046	x
*P*_3_	1.872	0.013	1.837	0.017	0.033	x
*Ac*_7_: *Frequency* *Range* (18.0–21.4)						
*P*_7_	1.850	0.017	1.814	0.025	0.022	x
*C*_*z*_	1.858	0.010	1.832	0.014	0.068	x
*PO*_*z*_	1.883	0.008	1.835	0.013	0.001	0.001
*P*_*z*_	1.867	0.010	1.841	0.013	0.027	x
*O*_*z*_	1.861	0.012	1.829	0.016	0.067	x
*O*_2_	1.867	0.012	1.828	0.019	0.032	x
*C*_1_	1.843	0.022	1.798	0.030	0.041	x
*P*_3_	1.867	0.011	1.832	0.018	0.049	x
*Ac*_8_: *Frequency* *Range* (21.0–24.6)						
*P*_7_	1.859	0.016	1.827	0.021	0.037	x
*P*_8_	1.868	0.011	1.846	0.011	0.094	x
*P*_3_	1.873	0.012	1.838	0.013	0.029	x
*P*_4_	1.877	0.009	1.854	0.010	0.052	0.040
*Ac*_9_: *Frequency* *Range* (24.2–28.0)						
*C*_1_	1.866	0.014	1.834	0.020	0.061	x
*Ac*_10_: *Frequency* *Range* (27.6–31.2)						
*AF*_4_	1.867	0.009	1.895	0.009	0.019	0.021
*Ac*_11_: *Frequency* *Range* (31.0–34.8)						
*C*_2_	1.874	0.009	1.90	0.010	0.047	x
*Ac*_12_: *Frequency* *Range* (34.4–38.2)						
*P*_7_	1.857	0.009	1.882	0.010	0.072	x

Utilizing this novel AC approach, we demonstrate that there is a significant increase in complexity during hypoxia across numerous EEG sites in the theta, alpha, and beta EEG frequency regions. The only significant decrease in complexity exists in the high frequencies of the gamma region. The sites that demonstrated this significant decrease in complexity never overlapped with the reported higher complexity EEG sites in the lower frequency regions. The rear left side of the brain, *P*_7_, *P*_3_, and *C*_1_ had the most consistent amount of significant activation complexities across frequency bands having 5, 3, and 4, respectively. In the introduction, we discussed how hypoxia has been hypothesized to cause neuronal isolation in past literature^11^. This concept was pictorially demonstrated in Fig. 1, where the time-frequency intensity peaks were more prominent for the functional isolation case.

We found that this AC method is not ideal, however, for small windowed segments of data. It is ideal for long-term trend analysis applications and has the potential to indicate small, subtle, anomalous patterns within the EEG spectral bands. Sample entropy and other temporal entropy measurements typically require a minimum of 100 data points or more^44^. The 10-minute segments that were analyzed only produced a mean of 431.2 peaks for each intensity frequency band, ${\widehat{\rho }}_{i}(n)$. When analyzing the number of peaks in each ${\widehat{\rho }}_{i}(n)$, for each EEG lead against the hypoxia and sea level cohorts, the one-sample t-test produced 18 significant p-values. The informative value in measuring how intensity is maximized and fluctuates is further supported. However, only 5 of the 18 significant p-values intersected with the 26 different statistically significant AC measurements. This alludes to the fact that it is not simply the amount of peaks but the timing of these peaks, and intensity may hold a very different meaning when it comes to analysis of complex brain dynamics. Therefore, how a band-limited intensity is sustained may provide valuable information regarding neuronal firing and indicators for disease.

### RQ3: classical methods

We hypothesize that classical EEG methods such as intensity (i.e., power) analysis can still produce valuable information, but that they provide an incomplete picture. Moreover, we specifically use spectral intensity analysis (SIA) since this method is the only approach in which we can compare effects on time, spectral bands, and intensity.

We discuss this hypothesis by first analyzing the changes in EEG intensity caused by hypoxia. Utilizing the filter banks’ summed intensity values for each 10-minute segment, we performed statistical one-sample t-tests. Due to the fact that electrical conductance can change from subject to subject and thereby alter the intensity values, each subject’s individual filter intensity values were normalized. When individual subject normalization was not applied, no significant difference was found. However, with proper normalization to account for electrical conductance changes, we found 26 filter intensity values across the 16 leads that were significantly different with one-sample t-tests when no multiple comparison correction (NMCC) was performed. A bootstrap t-test method was utilized to correct for multiple comparison correction (MCC) within the EEG analysis, which no single filter demonstrated significance between the hypoxic and non-hypoxic conditions. However, various correct methods could have been applied to adjust for multiple comparisons in which more conservative methods could greatly impact the significance and more liberal methods could potentially not impact initial result at all. Thus, we felt that the non-correction t-test still provides information on the trajectory of some leads that were close to being significant and still provides value to the reader.

Since our aim is not to directly discuss the effects of intensity and its relationship to hypoxia, but rather to determine the significant indicators that an intensity analysis provides, for brevity, we will only provide the results of significant EEG sites and their filters rather than the 768 means and standard errors associated with the intensity data (16 leads × 12 filters × 2 Conditions × 2 mean/SD).

*Spectra of No Change (Using NMCC):* The filters 3, 4, 10, and 11, which are associated to frequency bands (7.2–10.4 Hz), (10.0–12.8 Hz), (27.6–31.2 Hz), and (31.0–34.8 Hz), demonstrated no significant intensity change across any of the EEG electrodes for SIA. However, AC demonstrated significance for all four frequency bands, shown in Table 3. This demonstrates additional features that AC has extracted from the EEG signal.

*Spectra of Significant Increases (Using NMCC):* SIA did demonstrate significant increases in intensity only for Filters 1, 5, 6, 7, and 12 for the hypoxia cohort for a variety of EEG leads. More specifically, Filter 1 showed a significant increase in intensity for the *O*_2_ EEG site. Filters 1 and 5 showed an significant increase in intensity during Hypoxia for the *O*_2_ EEG site with p-values of 0.020 and 0.038, respectively. AC demonstrated no significance for Filter 1. However, for Filter 5, AC demonstrated increases for all the occipital recording sites (*O*_1_, *O*_*z*_, and *O*_2_) as well as other EEG sites. For Filter 6, SIA showed the largest change across EEG leads, exhibiting a significant increase in intensity during hypoxia for *PO*_*z*_, *P*_*z*_, *P*_8_, *O*_1_, *O*_*z*_, *O*_2_, *AF*_4_, *F*_2_, *C*_1_, and *P*_4_, with p-values of 0.0006, 0.007, 0.018, 0.015, 0.002, 0.024, 0.040, 0.039, 0.041, and 0.009, respectively. All of these sites, except for *O*_1_ and *C*_1_ are located on the right hemisphere. On the other hand, the AC measurement only reports significant increases on the left hemisphere (6 significant sites), demonstrating divergent findings across methods. For Filter 7, SIA also demonstrates significant increases in intensity for hypoxia for *PO*_*z*_, *F*_1_, *F*_2_, and *P*_4_, with p-values of 0.045, 0.030, 0.024, and 0.027. These recording sites are essentially the frontal lobe and left parietal lead. AC provided 8 significant sites, but only overlaps with *PO*_*z*_. The majority of AC are located in the posterior right part of the brain in the parietal and occipital recording sites for the 10–20 montage. For Filter 12, SIA exhibits a significant increase during hypoxia for *O*_*z*_, with a p-value of 0.035, and AC reports an increase for *P*_7_ during hypoxia. Overall, we can note that although both methods unanimously demonstrate significant increases with their respective method, there is very little to no overlap with regard to EEG recording sites.

*Spectra of Significant Decreases (Using NMCC):* Filters 2, 8, and 9 showed a decrease in intensity during hypoxia across various EEG locations. Filter 2 showed a decrease in intensity for *PO*_*z*_, *P*_*z*_, *O*_*z*_, and *P*_*z*_ with p-values of 0.038, 0.013, 0.0009, and 0.018, respectively. AC only reports *PO*_*z*_ as increased intensity during hypoxia. Filter 8 demonstrated a significant decrease during hypoxia for *O*_2_, *AF*_3_, and *AF*_4_, with respective p-values of 0.035, 0.047, and 0.018. We can also note an increased AC for the parietal recording site during hypoxia. Filter 9 also has *AF*_3_, and *AF*_4_, which exhibited a decrease in intensity with p-values of 0.029 and 0.026, respectively. AC only reported *C*_1_ as an increase during hypoxia.

*Results Summary:* In summary, we note that when comparing AC to SIA for NMCC the two methods are very divergent in their reported findings, specifically on the direction of the measurement (increasing vs decreasing), recording site of the brain, and spectral properties. These divergent results support the hypothesis that AC adds an additional dimensionality to the analysis. These SIA results share a resemblance to Papedelis’s work with hypoxia, where they reported an increase in spectral power^11^. One caveat was that the majority of the spectral intensity findings were on the right hemisphere, whereas Papadelis reported left hemisphere dominance^11^. However, our subjects utilized their left hands for the MATB tracking tasks whereas the subjects in Papadelis’s study used their right hands^11^. EEG asymmetries and cerebral lateralization in literature is well known and may explain the discrepancy between our results^11,45^. However, from the perspective of implementing MCC, the AC methodology demonstrates significant changes within the EEG signal during mild induction of hypoxia, unlike the standard spectral analysis.

### RQ4: comparing EEG methods for cognitive impairment

In order to formally compare spectral intensity and the new proposed approach coined, “activation complexity”, we compare the two approaches through evaluating it’s predictability to detect cognitive impairment. Literature has already shown that as the level of hypoxia becomes increasingly critical, a human’s cognitive impairment increases as well^46,47^. For this comparison, segmented instances of induced hypoxia were annotated into four different levels: *H*_1_) Completely non-hypoxic state (100–95% O2); *H*_2_) Indifferent hypoxia (95–85% O2); *H*_3_) Compensatory Hypoxia (75–85% O2) ;*H*_4_) Critical (Disturbance) (Less than 75% O2). Both methods share the exactly same dimensionality, sample size and predictive model (K-Nearest Neighbour approach), allowing for a fair comparative analysis to gauge how a specific feature set provides better predictive discriminators for cognitive impairment. The predictive performance for detecting the four levels of hypoxia using activation complexity (Table 4) verse spectral intensity (Table 5) are highlighted using confusion matrices with an accuracy (*Ac*) of 80.2 vs 71.3 %, respectively. These results demonstrate that Activation Complexity features covers more informative variance in the data and provides a superior predictive lift with KNN modeling. Thus, this comparison highlights that activation complexity provides information in characterizing cognitive impairment in which classical spectral intensity analysis can not provide.

**Table 4:** Confusion Matrix: Activation Complexity (*A*_*c*_ = 80.2%).

		Predicted			
		*H*_1_	*H*_2_	*H*_3_	*H*_4_
Actual	*H*_1_	**679**	32	29	2
	*H*_2_	47	**93**	25	1
	*H*_3_	62	29	**247**	11
	*H*_4_	8	2	11	**32**

**Table 5:** Confusion Matrix: Classical Spectral Power (*A*_*c*_ = 71.3%).

		Predicted			
		*H*_1_	*H*_2_	*H*_3_	*H*_4_
Actual	*H*_1_	**607**	47	84	4
	*H*_2_	51	**74**	40	1
	*H*_3_	54	44	**226**	25
	*H*_4_	8	1	16	**28**

## Conclusions

Our new complexity method for non-linear dynamic analysis for isolated EEG frequency bands and intensity analysis. This work added to the philosophy for decoding the amplitude and temporal dynamics has embedded information^48^ and how amplitude of the instantaneous frequency is related to unconsciousness^49^. However, our work demonstrates an elegant method that combines these philosophies to extract embedded information of the instantaneous frequency’s intensity (not amplitude) temporal dynamics and this relationships to impaired neuronal firing/neuronal isolation. This works supports the case for a new predictive EEG feature for hypoxia and opens up a novel avenue for analysis of diseases and physiological sensory changes.

More specifically, this method shows significant differences in hypoxic vs. non-hypoxic states, facilitating future analyses (specifically for diseases related to stroke, ischemia, and cognitive changes). We hypothesized that these methods are extracting features which manifest due to various forms of neuro-isolation, but to fully support this hypothesis, we require additional study designs. While EEG more directly measures the electrical activation of neurons, functional near infrared spectroscopy (fNIRS) can be used to detect local activity by measuring the hemodynamic response in the brain. As with functional magnetic resonance imaging (fMRI), measuring the hemodynamic response is a standard technique for quantifying brain activity based on neurovascular coupling. However unlike fMRI, fNIRS can be used passively while operationally-relevant, cognitively-engaging tasks are performed and without running costly trials^50^. Additionally, fNIRS can be used without the data acquisition and processing burdens of performing EEG source localization. The hemodynamic response, as measured by fNIRS, is heavily influenced by local activity in the capillary bed^51^ and vessels of diameter <1 mm^52^. Further, the neurovascular coupling relationship holds true where there is suppression of, or interference with, neuronal activity^53^. Thus, the use of fNIRS can supplement the newly proposed Activation Complexity measurements by quantifying local hemodynamic activity changes in the face of interference due to hypoxia. Further, the quantification of Activation Complexity as a more general measure of neuronal isolation in the brain would be supported by demonstrating a link between a decrease of blood oxygenation and an increase in Activation Complexity. If these global electrical and hemodynamic measurements are coupled, Activation Complexity has the potential of being a critical, fast, and low cost surrogate measurement for characterizing, and possibly detecting, numerous diseases such as mild localized strokes, cerebral ischemia, or brain trauma in which neuronal isolation plays an important part.

This work ultimately contributes an additional dimension of spectral and complexity analysis, opening the exploration of EEG signal for further explanatory analysis in the area of neurology and cognitive science. The ability to isolate the intensity of neuro-oscillations as a function of time allows for further explorations into not only the timing of peak intensity, but additional multi-variate features such as their trough and width dimensions. These additional complexity analyses can potentially address how these band-limited neuro-oscillations are sustained.
